# The association between oral carbohydrate intake before orthopedic surgery for osteoporotic fractures and outcomes in elderly patients

**DOI:** 10.1186/s13018-023-04458-1

**Published:** 2023-12-14

**Authors:** Jing Zhu, Xue-qin Jin, Xiao-yan Li, Li Sun, Yuan Peng

**Affiliations:** 1 Department of Nursing, Affiliated Kunshan Hospital of Jiangsu University, Suzhou, Jiangsu China; 2 Intensive Care Unit, Affiliated Kunshan Hospital of Jiangsu University, No. 566 East of Qianjin Road, Suzhou, Jiangsu China

**Keywords:** Carbohydrate, Osteoporotic fracture, Mortality, Length of hospital stay, Hospitalization cost, Elderly patients

## Abstract

**Background:**

Oral carbohydrate (CHO) intake is a safe method with effective clinical results in various surgical patients before surgery. Nevertheless, due to a lack of adequate clinical data, it is not frequently utilized in older patients undergoing orthopedic surgery for osteoporotic fractures (OPFs), especially in China. The purpose of the present study was to examine the relationship between preoperative oral CHO consumption and outcomes in elderly patients undergoing surgical treatment for OPFs.

**Methods:**

This retrospective cohort study was conducted at a single Chinese institution and included a total of 879 elderly patients (median age: 71 years; range: 50–99 years) who underwent OPF surgery. Various exclusion criteria were established as follows: (a) the necessity for urgent surgical intervention; (b) the existence of hypoglycemia, hyperglycemia, or diabetes mellitus with blood glucose levels lower than 2.8 mmol/L; (c) a medical history of gastrointestinal motility disorders or delayed gastric emptying; (d) the utilization of local anesthesia; (e) a Charlson comorbidity index (CCI) score over 2; and (f) an American Society of Anesthesiologists (ASA) score exceeding 3. After propensity score (PS) matching, 264 patients from each cohort were included in the analysis. The primary outcome was the all-cause mortality rate within 60 days post-surgery, while secondary outcomes included the length of hospital stay (LOS), hospitalization costs, intraoperative and postoperative blood transfusions, and the incidence of postoperative nausea and vomiting (PONV) and aspiration. The relationship between preoperative oral CHO intake and outcomes was evaluated using multivariate regression analysis.

**Results:**

After PS matching, preoperative oral CHO intake was negatively associated with 60-day mortality in the fully adjusted model (odds ratio 0.35; 95% confidence interval 0.12–0.97; *P*-value: 0.04). Patients who received preoperative oral CHO intake also had a shorter LOS and lower hospitalization costs than those who did not receive CHO intake. However, none of the models showed a significant association between CHO intake and PONV or blood transfusion risk. Furthermore, no cases of aspiration were observed in either cohort.

**Conclusions:**

Preoperative oral CHO intake may be associated with reduced mortality risk and improved outcomes in elderly patients undergoing surgical treatment for OPFs. However, it is important to acknowledge the limitations of our study, including its retrospective nature, potential unmeasured confounding variables, the small sample size, incomplete data on important variables such as duration of surgery and inflammatory markers, and the limited generalizability due to the participation of only one institution. Future research with larger sample sizes and a broader range of events is warranted to validate and enhance the validity of our findings, particularly in assessing long-term results and understanding the underlying mechanisms.

## Introduction

Osteoporosis is a common condition observed in women who have reached the postmenopausal stage, characterized by the loss of microarchitectural structure and a decrease in bone mass [1]. Fragility fractures, also known as osteoporotic fractures (OPFs), are frequent in older people, with lifetime risks for women of 40–50% and men of 12–22% [2]. Individuals with OPFs who are 65 years of age or older are more likely to face negative health outcomes such as reduced life expectancies, persistent impairment, extended hospitalizations, and decreased mobility [3–5]. Especially when the lower extremity is affected, surgical intervention is necessary for most OPFs [4]. Most individuals acquire pain relief and a higher degree of function following surgical stabilization than conventional therapy [6].

Fasting from midnight before general anesthesia introduction is often recommended for older surgical patients to minimize the amount and acidity of stomach contents during operation and, therefore, lower the risk of pulmonary aspiration [7]. However, the empirical evidence supporting this practice is limited [8]. Only six of the 22 randomized controlled trials (RCTs) in elective gynecological and general surgery investigated the incidence of aspiration, and no aspiration events were reported, according to a Cochrane analysis of the studies [9]. Additionally, there was no discernible difference between individuals in the fasting cohort and those who were permitted clear fluids up to 2 h before anesthesia administration regarding the amount or pH of gastric content [9]. Clear liquids, including water and black coffee, may be ingested up to 2 h before surgery, necessitating general anesthesia and a light solid meal can be taken up to 6 h before surgery, according to current fasting guidelines and improved postoperative recovery [8, 10–12].

A catabolic condition induced by surgical trauma can result in postoperative hyperglycemia and other physiological abnormalities that may affect recovery. This state is characterized by accelerated protein breakdown and insulin resistance [13]. Preoperative carbohydrate (CHO) drinks may reduce the adverse effects of overnight fasting by minimizing postoperative insulin resistance, hyperglycemia, and the need for insulin treatment while maintaining skeletal and cardiac muscle function. These drinks have a low osmolality and about 12% CHO content and are primarily based on maltodextrins and some salt [10]. Preoperative CHO beverages may reduce preoperative discomfort, stress, headache, vomiting, nausea after surgery, pain, and inflammatory reaction without raising the risk of pulmonary aspiration [14, 15].

However, two RCTs examining its impact on glucose management in patients undergoing spinal surgery failed to show an advantage, raising doubt about the clinical utility of preoperative CHO loading in orthopedic surgery [16, 17]. Oral CHO intake did not reduce postoperative insulin resistance, according to research on patients who suffered from proximal femoral fractures treated with closed reduction and internal fixation [18]. Finally, similar studies evaluating outcomes in elderly surgical patients only consider postoperative indicators such as subjective feelings (anxiety, vomiting, nausea, thirst, and hunger), insulin resistance, and serum glucose levels but do not investigate the impact of CHO on mortality in this population.

We conducted a real-world study to determine whether preoperative oral CHO intake could increase postoperative outcomes in older patients receiving orthopedic surgery for OPFs to further our understanding of this problem. We hypothesized that CHO intake before surgery would result in better postoperative outcomes, particularly regarding short-term mortality, than those who received plain water.

## Material and methods

### Study design and population

The present study examines prospectively obtained information from the osteoporotic fracture registry system (OPFRS) at the Affiliated Kunshan Hospital of Jiangsu University (AKHJU). AKHJU is recognized as a prominent tertiary-level healthcare institution in the Kunshan area of Jiangsu, China. The OPFRS functions as an extensive repository that systematically collects pertinent clinical data regarding fractures proactively caused by osteoporosis. The OPFRS covers all OPFs of this hospital, ensuring a comprehensive representation of the patient population and their associated data. The present study utilizes the database mentioned above to examine a range of features associated with OPFs and their subsequent repercussions. We acquired electronic records of patients aged 50 years and over who underwent surgical treatment for an OPF in our institution between January 1, 2020, and March 31, 2021 (totaling 1043 patients). The choice of this study period was based on the availability of electronic records and the feasibility of data collection within a reasonable timeframe. The selected period allowed us to capture a substantial number of patients and ensure an adequate sample size for analysis.

**Fig. 1:**
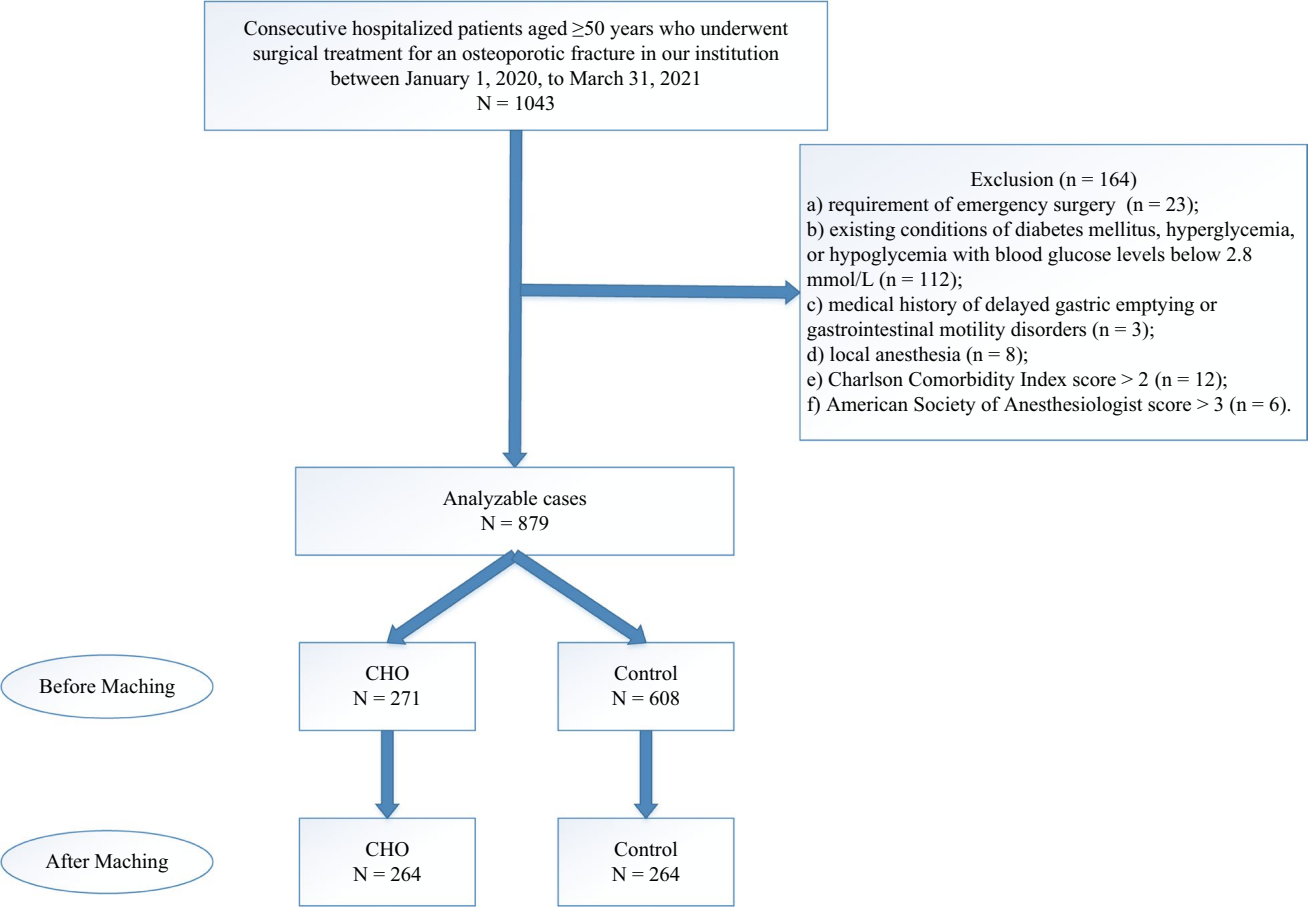
Flowchart of the Study Design and Methodology. CHO, carbohydrate

The fracture sites qualified for this study were the wrist, proximal humerus, hip, or vertebra. The OPF diagnosis was determined in compliance with the standards established by the 2017 Chinese Guidelines for OPF diagnosis and treatment [19]. A subset of 164 patients was removed from the analysis due to several predetermined exclusion criteria: (a) requirement of emergency surgery (*n* = 23); (b) existing conditions of hypoglycemia, hyperglycemia, or diabetes mellitus [18, 20], with blood glucose levels below 2.8 mmol/L (*n* = 112); (c) medical history of gastrointestinal motility disorders or delayed gastric emptying (*n* = 3); (d) use of local anesthesia (*n* = 8); (e) Charlson comorbidity index (CCI) score exceeding 2 (*n* = 12); and (f) American Society of Anesthesiologists (ASA) score surpassing 3 (*n* = 6). Ultimately, the analysis encompassed 879 patients (see Fig. 1).

The patient population was divided into two distinct cohorts: the CHO cohort, who were administered a preoperative oral CHO beverage, and the control cohort, who were given plain water. The decision regarding CHO administration was entrusted to the attending orthopedic surgeons for each patient. We strictly followed the Declaration of Helsinki Ethical Principles to assure complete conformity to ethical standards, and our regional institutional review board provided authorization (approval number: 2022-06-035; date: 2022-05-25) for this study. Furthermore, we procured informed consent from all participants before initiating treatment procedures.

### Preoperative CHO loading and water-only fasting protocol

**Fig. 2:**
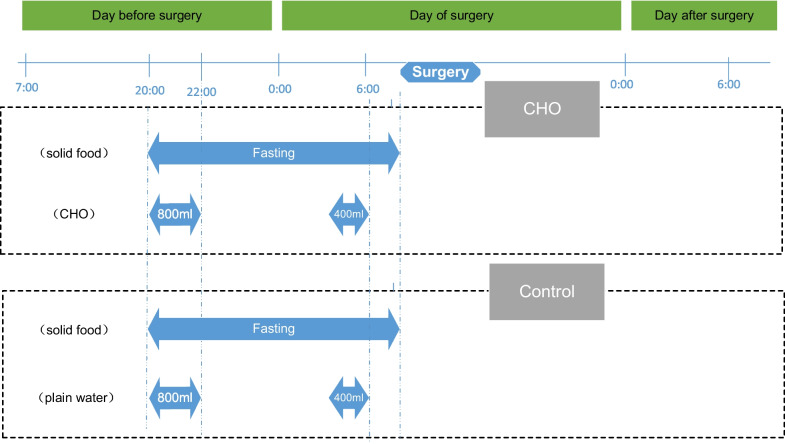
Preoperative CHO Loading and Water-only Fasting Protocol of the Study. CHO, carbohydrate

Figure 2 illustrates the preoperative CHO loading and water-only fasting protocol used in our study. Patients in the CHO cohort received a liquid mixture containing 12.5 g of CHO (fructose, 1.2 g and maltodextrin, 11.3 g) per 100 mL [245 mosml/L; 500 kcal/L (215 kJ)] (Suqian, Zheng Da Feng Hai Pharmaceutical Co Ltd, Jiangsu, China). They were advised to drink 800 mL of this mixture between 20:00 and 22:00 the evening before surgery and an additional 400 mL 2 h before. Solid food intake was prohibited 12 h before the surgery, and other fluids or solutions were strictly off-limits. Surgeries were scheduled between 8:00 and 12:00. On the other hand, the control cohort was directed to drink plain water while adhering to the same timing and amount restrictions as the CHO cohort.

### Outcomes

The primary outcome of this investigation was the all-cause mortality rate within 60 days post-surgery. The requisite mortality data were obtained and authenticated through the Jiangsu Provincial Population Mortality Registry System, ensuring dependable and precise insight into patient outcomes. The secondary outcomes included the length of hospital stay (LOS), hospitalization costs, intraoperative and postoperative blood transfusions, and the occurrence rate of postoperative nausea and vomiting (PONV) and aspiration.

### Covariates

All relevant information on covariates was procured directly from the OPFRS database at AKHJU, negating the need for manual extraction from individual patient records or charts. The information gathered included data on patient demographics, history of fractures, current diagnoses, co-existing medical conditions, results from preoperative laboratory tests, and surgical and anesthesia-related specifics. The ASA score and the CCI [21] were used to determine the patient's overall physical health and any pre-existing medical disorders. The principal anesthesiologist overseeing the case examined and recorded the ASA ratings [22]. According to Quan et al. [23], the CCI was estimated using ICD-10 codes based on diagnoses recorded within the year preceding the surgery [24]. CCI was calculated algorithmically utilizing information obtained from electronic medical records. Former or past smokers within the previous 12 months were included in the definition of smoking.

Similarly, drinking was defined as consuming alcohol at least once weekly for 12 months. Any fracture occurring before the primary fracture date within the past 14 years [25] was documented as a prior fracture. The fracture locations under consideration included wrist, proximal humerus, hip, and spine fractures.

### Statistical analysis

Demographic and clinical data for patients were presented using either the median (first quartile [Q1] to third quartile [Q3]) or mean ± standard deviation (SD), depending on the distribution of the data. The Mann–Whitney *U*-test was employed to evaluate non-normally distributed data, whereas independent two-tailed *t*-tests were utilized to compare regularly distributed data. Categorical data were given as frequency (%), and Chi-squared tests were used to analyze differences. Fisher's exact test was employed instead of the Chi-squared test if the assumptions were unmet.

To address the differences in baseline characteristics between the two patient cohorts (Table 1), propensity score (PS) matching was utilized to identify a group of patients with comparable baseline characteristics. The PS represents the conditional probability of a specific exposure given a particular set of baseline covariates. A non-parsimonious multivariate model [26] was used to predict the PS, with and without preoperative oral CHO as the dependent variable and all baseline characteristics (Table 1) as covariates. A 1:1 matching procedure was employed without replacement using a greedy matching algorithm, with a caliper width set to 0.02 of the SD of the PS logit. Standardized differences were calculated for all baseline covariates before and after matching to assess pre-match imbalance and post-match balance. A standardized difference of < 0.10 indicated a relatively small imbalance in the covariate [27].

**Table 1:** Baseline Characteristics before and after Propensity Score Matching

Characteristics	Before matching				After matching			
	Control	CHO	Standardized difference^a^	*P*-value	Control	CHO	Standardized difference^a^	*P*-value
	(*N* = 608)	(*N* = 271)			(*N* = 264)	(*N* = 264)		
	Mean (SD) Median (*Q*1–*Q*3)/*N* (%)				Mean (SD) Median (*Q*1–*Q*3)/*N* (%)			
Age	69.90 (11.63) 70.00 (61.00–79.00)	74.75 (9.76) 74.00 (67.00–82.00)	0.45 (0.31, 0.60)	< 0.001	75.76 (10.43) 76.50 (68.00–84.00)	74.45 (9.62) 73.50 (66.00–82.00)	0.13 (− 0.04, 0.30)	0.135
BMI	22.95 (3.38) 22.86 (20.75–25.13)	23.11 (3.41) 23.01 (20.52–25.39)	0.05 (− 0.09, 0.19)	0.506	22.75 (3.49) 22.49 (20.28–25.26)	23.06 (3.41) 22.86 (20.49–25.35)	0.09 (− 0.08, 0.26)	0.305
Serum sodium, mmol/L	140.66 (2.73) 140.80 (139.10–142.40)	140.85 (3.01) 140.90 (139.10–142.75)	0.07 (− 0.08, 0.21)	0.349	140.58 (2.88) 140.85 (139.00–142.40)	140.89 (2.98) 140.90 (139.10–142.70)	0.10 (− 0.07, 0.27)	0.234
Serum potassium, mmol/L	3.89 (0.45) 3.89 (3.60–4.13)	3.87 (0.43) 3.87 (3.60–4.08)	0.04 (− 0.10, 0.18)	0.604	3.91 (0.45) 3.92 (3.65–4.15)	3.86 (0.41) 3.87 (3.60–4.08)	0.11 (− 0.06, 0.28)	0.202
Serum calcium, mmol/L	2.20 (0.13) 2.20 (2.12–2.29)	2.20 (0.12) 2.21 (2.13–2.27)	0.02 (− 0.13, 0.16)	0.818	2.20 (0.14) 2.21 (2.12–2.29)	2.20 (0.12) 2.21 (2.13–2.27)	0.01 (− 0.16, 0.18)	0.905
Serum magnesium, mmol/L	0.89 (0.10) 0.89 (0.82–0.95)	0.88 (0.11) 0.89 (0.82–0.95)	0.05 (− 0.09, 0.19)	0.489	0.88 (0.10) 0.88 (0.82–0.95)	0.88 (0.11) 0.89 (0.82–0.95)	0.01 (− 0.16, 0.18)	0.928
Serum phosphate, mmol/L	1.07 (0.21) 1.07 (0.94–1.20)	1.07 (0.19) 1.06 (0.95–1.19)	0.04 (− 0.11, 0.18)	0.61	1.09 (0.21) 1.10 (0.96–1.21)	1.07 (0.19) 1.06 (0.95–1.19)	0.12 (− 0.05, 0.29)	0.184
Hemoglobin, g/L	126.12 (20.31) 129.00 (114.50–141.00)	124.70 (18.35) 127.00 (113.10–138.80)	0.07 (− 0.07, 0.22)	0.322	125.74 (21.37) 129.00 (113.00–141.00)	124.88 (17.85) 127.00 (113.80–138.00)	0.04 (− 0.13, 0.21)	0.618
Platelet count, × 10^9^/L	175.98 (64.45) 166.00 (136.00–211.00)	177.50 (69.59) 166.00 (130.00–214.95)	0.02 (− 0.12, 0.17)	0.754	171.61 (61.97) 162.00 (134.00–205.00)	178.20 (70.02) 166.50 (130.00–215.00)	0.10 (− 0.07, 0.27)	0.253
Fasting blood glucose, mmol/L	5.45 (0.73) 5.51 (5.05–5.89)	5.47 (0.73) 5.60 (5.08–5.90)	0.03 (− 0.11, 0.18)	0.662	5.45 (0.71) 5.50 (5.05–5.92)	5.47 (0.73) 5.59 (5.07–5.90)	0.03 (− 0.14, 0.20)	0.748
Serum albumin, g/L	39.93 (4.47) 40.10 (37.08–42.92)	39.72 (3.88) 40.10 (37.40–42.20)	0.05 (− 0.09, 0.20)	0.488	39.95 (4.52) 40.20 (36.80–42.92)	39.68 (3.87) 40.10 (37.30–42.20)	0.06 (− 0.11, 0.23)	0.461
Neutrophil count, × 10^9^/L	6.55 (3.15) 5.88 (4.30–8.00)	6.57 (2.82) 6.10 (4.56–8.26)	0.01 (− 0.14, 0.15)	0.917	6.56 (3.19) 5.90 (4.40–8.00)	6.53 (2.77) 6.05 (4.51–8.23)	0.01 (− 0.16, 0.18)	0.918
Lymphocyte count, × 10^9^/L	1.26 (0.53) 1.20 (0.90–1.60)	1.25 (0.56) 1.20 (0.90–1.53)	0.02 (− 0.13, 0.16)	0.801	1.28 (0.52) 1.20 (0.90–1.60)	1.26 (0.57) 1.20 (0.90–1.56)	0.05 (− 0.12, 0.22)	0.576
Monocyte count, × 10^9^/L	0.55 (0.49) 0.50 (0.40–0.70)	0.52 (0.27) 0.50 (0.38–0.63)	0.07 (− 0.07, 0.22)	0.366	0.51 (0.25) 0.48 (0.35–0.64)	0.52 (0.27) 0.50 (0.40–0.63)	0.02 (− 0.15, 0.19)	0.783
ALT, U/L	23.94 (21.53) 20.00 (14.00–28.00)	23.61 (16.96) 19.00 (15.00–26.00)	0.02 (− 0.13, 0.16)	0.824	25.35 (27.76) 20.00 (14.75–28.00)	23.46 (16.90) 19.00 (15.00–26.00)	0.08 (− 0.09, 0.25)	0.345
AST, U/L	26.25 (19.58) 23.00 (18.00–29.00)	25.59 (16.30) 22.00 (19.00–27.50)	0.04 (− 0.11, 0.18)	0.629	27.12 (26.02) 23.00 (18.00–29.00)	25.38 (15.79) 22.00 (19.00–27.25)	0.08 (− 0.09, 0.25)	0.355
Serum creatinine, μmol/L	66.98 (22.92) 63.00 (54.00–76.00)	67.30 (22.69) 64.00 (55.00–76.00)	0.01 (− 0.13, 0.16)	0.845	68.91 (24.80) 65.00 (55.00–78.00)	66.31 (19.70) 63.00 (54.75–74.25)	0.12 (− 0.05, 0.29)	0.182
Serum urea nitrogen, mmol/L	6.08 (2.32) 5.60 (4.61–6.90)	5.95 (2.30) 5.60 (4.48–6.82)	0.06 (− 0.09, 0.20)	0.441	6.22 (2.45) 5.70 (4.80–7.10)	5.87 (2.11) 5.57 (4.45–6.80)	0.15 (− 0.02, 0.32)	0.081
Serum uric acid, μmol/L	286.99 (91.67) 282.00 (223.25–344.00)	286.14 (87.16) 276.00 (232.50–332.00)	0.01 (− 0.13, 0.15)	0.898	287.37 (90.65) 282.00 (222.50–342.00)	283.92 (85.93) 275.50 (229.75–328.00)	0.04 (− 0.13, 0.21)	0.654
Sex			0.19 (0.04, 0.33)	0.01			0.02 (− 0.16, 0.19)	0.858
Female	422 (69.41%)	164 (60.52%)			161 (60.98%)	163 (61.74%)		
Male	186 (30.59%)	107 (39.48%)			103 (39.02%)	101 (38.26%)		
Smoking			0.03 (− 0.11, 0.17)	0.683			0.01 (− 0.16, 0.18)	0.874
No	557 (91.61%)	246 (90.77%)			243 (92.05%)	242 (91.67%)		
Yes	51 (8.39%)	25 (9.23%)			21 (7.95%)	22 (8.33%)		
Drinking			0.10 (− 0.04, 0.25)	0.143			0.00 (− 0.17, 0.17)	1
No	580 (95.39%)	252 (92.99%)			247 (93.56%)	247 (93.56%)		
Yes	28 (4.61%)	19 (7.01%)			17 (6.44%)	17 (6.44%)		
Fracture site			0.19 (0.05, 0.34)	0.069			0.21 (0.04, 0.38)	0.115
Spine	288 (47.37%)	107 (39.48%)			98 (37.12%)	107 (40.53%)		
Hip	230 (37.83%)	107 (39.48%)			128 (48.48%)	103 (39.02%)		
Proximal humerus	17 (2.80%)	11 (4.06%)			7 (2.65%)	11 (4.17%)		
Wrist	73 (12.01%)	46 (16.97%)			31 (11.74%)	43 (16.29%)		
ASA score			0.19 (0.04, 0.33)	0.031			0.14 (− 0.03, 0.31)	0.282
1	93 (15.30%)	38 (14.02%)			31 (11.74%)	38 (14.39%)		
2	432 (71.05%)	177 (65.31%)			189 (71.59%)	172 (65.15%)		
3	83 (13.65%)	56 (20.66%)			44 (16.67%)	54 (20.45%)		
CCI			0.15 (0.01, 0.29)	0.17			0.07 (− 0.10, 0.24)	0.735
0	533 (87.66%)	244 (90.04%)			242 (91.67%)	237 (89.77%)		
1	55 (9.05%)	24 (8.86%)			20 (7.58%)	24 (9.09%)		
2	20 (3.29%)	3 (1.11%)			2 (0.76%)	3 (1.14%)		
Prior fracture			0.19 (0.05, 0.33)	0.012			0.02 (− 0.15, 0.19)	0.806
No	479 (78.78%)	233 (85.98%)			224 (84.85%)	226 (85.61%)		
Yes	129 (21.22%)	38 (14.02%)			40 (15.15%)	38 (14.39%)		
Anesthesia			0.19 (0.05, 0.34)	0.03			0.15 (− 0.02, 0.32)	0.229
General anesthesia	322 (52.96%)	118 (43.54%)			119 (45.08%)	117 (44.32%)		
Spinal anesthesia	195 (32.07%)	100 (36.90%)			108 (40.91%)	96 (36.36%)		
Brachial plexus block anesthesia	91 (14.97%)	53 (19.56%)			37 (14.02%)	51 (19.32%)		
Hypertension			0.04 (− 0.10, 0.18)	0.569			0.01 (− 0.16, 0.18)	0.924
No	410 (67.43%)	188 (69.37%)			184 (69.70%)	185 (70.08%)		
Yes	198 (32.57%)	83 (30.63%)			80 (30.30%)	79 (29.92%)		

*CHO* carbohydrate, *BMI* body mass index, *SD* standard deviation, *CI* confidence interval, *ASA* American Society of Anesthesiologists, *CCI* Charlson comorbidity index, *AST* serum aspartate aminotransferase, and *ALT* serum alanine transaminase

^a^Standardized differences of < 0.10 for a given covariate indicate a relatively small imbalance

We used multivariate linear regression analysis after matching to assess the independent relationship between CHO and outcomes. We displayed the results of the unadjusted (Model 1), minimally adjusted (Model 2), and completely adjusted (Model 3) analyses simultaneously following the advice of the STROBE declaration. First, we used variance inflation factor (VIF) analysis to diagnose collinearity covariance. Next, we assessed the need for covariance adjustment based on the following: Criteria 1, a covariate was introduced to the basic model or removed from the full model, and the matched odds ratio (OR) was altered by a minimum of 10%; Criteria 2: Criteria 1 or a covariate *P*-value of < 0.1, based on the univariate model [28]. Hence, in the case of fully adjusted models, Model 2 was established according to Criteria 1, and Model 3 utilized Criteria 2.

Furthermore, we did subgroup analyses by classifying numerous factors to assess the robustness of our findings and potential differences within subgroups. We used the likelihood ratio test (LRT) to compare the associations and modifications observed in the subgroups. In order to discover effect modification, we investigated possible interactions between CHO and the variables. We used the Wald test to determine the significance of the interactions, with a significance threshold of 0.05. The R program (version 4.2.0, available at http://www.r-project.org) and EmpowerStats (http://www.empowerstats.com, X&Y Solutions, Inc., Boston, MA) was used for all statistical analyses. Statistical significance was defined as a two-sided *P*-value less than 0.05.

## Results

### Patient baseline features

This study comprised a total of 879 consecutive patients who underwent surgical therapy for an OPF at our facility. The control cohort had 608 patients, while the CHO cohort included 271 patients before matching. Following matching, there were 264 patients in each cohort. Figure 1 illustrates the matching procedure.

Concerning the control and treatment cohorts that received preoperative oral CHO consumption, Table 1 compares the baseline features of the study participants before and after PS matching. The baseline characteristics of the two cohorts, including sex, age, fracture site, ASA score, CCI score, prior fracture, and anesthesia, were significantly different before matching. However, after PS matching, the two cohorts were more balanced regarding sex, CCI score, and other clinical factors, with standardized differences of less than 0.1 for most variables.

The results suggest that PS matching was successful in reducing the impact of confounding factors and improving the comparability of the two cohorts. Specifically, the number of patients in the CHO cohort decreased from 271 to 264 after matching, and the number of patients in the control cohort also reduced from 608 to 264. The improvement in balance after matching was most evident in age and sex. The mean age of the CHO cohort before matching was 74.8, while the control cohort had a mean age of 70.0. When the two cohorts were compared, the mean ages were 74.5 and 75.8, respectively, with a normalized difference of 0.13 that was not statistically significant (*P*-value = 0.14). The same trend was observed for sex, with a standardized difference of 0.02 after matching.

### Primary outcome

After matching, seven out of 264 patients (2.7%) in the CHO cohort and 19 out of 264 patients (7.2%) in the control cohort died within 60 days after surgery, indicating a potential benefit of CHO intake. Table 2 presents the results of multivariate regression analysis, demonstrating a significant negative association between CHO and mortality in all three models. The crude unadjusted analysis (Model 1) revealed a significant association between CHO and mortality (OR 0.35; 95% C, 0.15–0.85; *P*-value = 0.02), while Model 2 and Model 3, which adjusted for various confounding factors, also showed a significant association between CHO and mortality (OR 0.37; 95% CI 0.14–0.97; *P*-value = 0.04 and OR 0.35; 95% CI 0.12–0.97; *P* = 0.04, respectively). These findings suggest that CHO intake may be associated with a reduced mortality risk after surgery, regardless of adjustment.

**Table 2:** The Association Between CHO and Outcomes in Different Models

Outcomes	Model 1^a^	Model 2^b^	Model 3^c^
Mortality, OR (95% CI) *P*-value	0.35 (0.15, 0.85) 0.020	0.37 (0.14, 0.97) 0.044	0.34 (0.12, 0.95) 0.039
LOS, *β* (95% CI) *P*-value, days	− 1.65 (− 2.42, − 0.88) < 0.001	− 1.44 (− 2.19, − 0.69) < 0.001	− 1.36 (− 2.11, − 0.61) < 0.001
Hospitalization cost, *β* (95% CI) *P*-value, U$	− 543.26 (− 909.74, − 176.78) 0.004	− 543.26 (− 909.74, − 176.78) 0.004	− 504.42 (− 872.49, − 136.35) 0.008
Blood transfusion, OR (95% CI) *P*-value	0.60 (0.32, 1.14) 0.118	0.82 (0.41, 1.63) 0.564	0.84 (0.42, 1.69) 0.627
PONV, OR (95% CI) *P*-value	1.00 (0.35, 2.89) 1.000	1.22 (0.41, 3.62) 0.721	1.10 (0.36, 3.31) 0.870

*OR* odds ratio, *CI* confidence interval, *CHO* carbohydrate, *BMI* body mass index, *LOS* length of stay, and *PONV* postoperative nausea and vomiting

^a^No adjustment

^b^Adjusted for age, sex, fracture site, and serum magnesium

^c^Adjusted for Model 2 plus BMI, anesthesia, serum potassium, serum creatinine, and the propensity score

### Secondary outcomes

In addition to mortality, Table 2 describes the association between CHO intake and other secondary outcomes. The results show that CHO intake significantly reduced LOS and hospitalization costs in all three models. The adjusted β coefficients for LOS ranged from − 1.36 to − 1.65, indicating that CHO intake was associated with a reduction in LOS by approximately 1.36–1.65 days. According to the corrected β coefficients for hospitalization costs, which ranged from − 504.42 to − 543.26, CHO consumption was correlated with a decrease in costs of about 504.42–543.26 US dollars. However, neither of the models was a connection between CHO consumption and the risk of PONV or blood transfusion (all *P*-values > 0.05). Furthermore, no cases of aspiration were observed in either cohort.

### Subgroup analyses

We further performed subgroup analyses based on the nine factors included in the fully adjusted multivariate regression model to explore the connection between CHO intake and mortality. The results of these analyses confirmed that the observed association between CHO intake and mortality was strong, even in the presence of possible confounders. The subgroup analyses (see Table 3) revealed no significant interactions between CHO intake and any of the nine covariates (all *P*-values for interaction > 0.05), indicating that the association between CHO intake and mortality was consistent across all subgroups. These findings provide further evidence supporting the robustness of the association between CHO intake and mortality in surgical patients.

**Table 3:** Subgroup Analyses of the Association between CHO and Mortality

Subgroup	*N*	OR (95% CI) *P*-value	*P*-value for interaction
Sex			0.97
Female	324	0.30 (0.05, 1.65) 0.1650	
Male	204	0.28 (0.07, 1.22) 0.0911	
Age tertile			0.61
Low	159	0.00 (0.00, Inf) 0.9998	
Middle	180	110.06 (0.00, Inf) 0.9999	
High	189	0.27 (0.09, 0.86) 0.0266	
BMI tertile			0.06
Low	175	0.87 (0.22, 3.48) 0.8412	
Middle	177	0.03 (0.00, 0.55) 0.0177	
High	176	0.00 (0.00, Inf) 0.9985	
Fracture site			0.78
Spine	205	0.03 (0.00, 5.20) 0.1800	
Hip	231	0.32 (0.10, 0.99) 0.0475	
Proximal humerus	18	1.00 (0.00, Inf) 1.0000	
Wrist	74	1.00 (0.00, Inf) 1.0000	
Anesthesia			0.95
General anesthesia	236	0.41 (0.06, 3.00) 0.3792	
Spinal anesthesia	204	0.28 (0.08, 1.00) 0.0499	
Brachial plexus block anesthesia	88	1.00 (0.00, Inf) 1.0000	
Potassium tertile			0.36
Low	174	inf. (0.00, Inf) 0.9930	
Middle	173	0.07 (0.01, 0.73) 0.0265	
High	181	0.53 (0.09, 3.08) 0.4831	
Magnesium tertile			0.56
Low	163	0.37 (0.01, 10.62) 0.5613	
Middle	184	0.03 (0.00, 3.91) 0.1624	
High	181	0.36 (0.09, 1.43) 0.1444	
Serum creatinine tertile			0.16
Low	176	0.66 (0.19, 2.37) 0.5268	
Middle	172	0.00 (0.00, Inf) 0.9808	
High	180	0.00 (0.00, Inf) 0.9948	

Adjusted for age, sex, fracture site, serum magnesium, BMI, anesthesia, serum potassium, and serum creatinine except the subgroup variable

*OR* odds ratio, *CI* confidence interval, *CHO* carbohydrate, and *BMI* body mass index

## Discussion

CHO administration is a safe method with effective clinical results prior to elective surgery [29]. However, due to a lack of appropriate clinical evidence, this approach is not frequently employed in older patients having orthopedic surgery for OPFs, particularly in China. The current research explored the relationship between older patients getting surgical therapy for OPFs and their preoperative oral CHO intake and results. We found that preoperative oral CHO intake may be linked with a reduced risk of short-term mortality, shorter LOS, and lower hospitalization costs.

The finding that preoperative oral CHO intake reduces mortality risk is consistent with the previous studies in other surgical populations, such as patients undergoing cardiac surgery [30]. One possible explanation for this association is that CHO intake may reduce insulin resistance [31]. Insulin resistance is a metabolic state in which the liver and peripheral tissues, mainly muscles, become less responsive to insulin. Approximately 85% of people with type 2 diabetes and 20% of those without diabetes have biochemically characterized insulin resistance [32]. Insulin resistance is primarily of muscle origin, rapidly develops during the perioperative period [33], and lasts long (weeks) after surgery. It appears to be a reaction to trauma and decreased energy intake, at least in part. The severity of insulin resistance is directly proportional to the magnitude of the surgical insult and is also associated with the development of postoperative complications [34]. In addition to many other metabolic impacts, insulin resistance also results in impaired muscle CHO oxidation, increased muscle catabolism, a negative nitrogen balance, decreased muscle mass [35], and diminished muscular strength [36]. Oral CHO loading has been shown to reduce insulin resistance by roughly 50% in elective orthopedic, colorectal, and laparoscopic surgeries [37], and the effect appears to be long-lasting [31, 38, 39]. Reducing insulin resistance through CHO intake may have several benefits for surgical patients. Studies have shown that lowering insulin resistance can improve glucose control and reduce the risk of complications such as infections, wound healing issues, and prolonged hospital stays [33]. Improved glucose control has also been associated with better surgical outcomes, including reduced mortality rates [40].

The second possible mechanism that may explain the reduced risk of mortality associated with preoperative oral CHO intake in surgical patients is the preservation of muscle mass. Earlier studies have revealed that after a hip fracture, there is an early and sustained decrease in muscle mass [41]. Muscle wasting can lead to decreased mobility, increased risk of falls, and other negative outcomes, further increasing mortality risk. CHO loading, which includes putting patients into surgery in a “metabolically fed state,” on the other hand, can help minimize protein and muscle loss after surgery [42]. By consuming CHO before surgery, patients can provide the body with energy not derived from protein breakdown, which can help spare muscle tissue. Ingestion of CHO can also stimulate the release of insulin, an anabolic hormone that promotes protein synthesis and avoids muscle degradation. This can help to maintain muscle mass and to prevent muscle wasting in the postoperative period.

The third possible mechanism is the improvement of immune function. Surgery and trauma can temporarily impair the immune system, which increases the risk of infections and other complications. Preoperative CHO intake has been shown to lessen the harmful impact of surgery on monocytes' expression of the human leukocyte antigen (HLA)-DR [43]. This is significant because a decrease in HLA-DR expression on monocytes after surgery has been associated with increased postoperative infectious complications [44]. Therefore, improving immune function through preoperative oral CHO intake may be a promising strategy for reducing the risk of postoperative infections and other complications, improving overall health outcomes, and reducing mortality risk in surgical patients.

The inclusion of magnesium, potassium, and creatinine in the model is justified based on their clinical significance and potential influence on the desired outcomes. The variables mentioned above pertain to standard laboratory tests on patients admitted to a hospital setting. Magnesium is pivotal in diverse physiological processes, whereas potassium is required for optimal cellular functioning, particularly in cardiac and muscular tissues [45, 46]. Creatinine is widely employed as a biomarker for the evaluation of renal function [47]. By integrating these factors, researchers can examine their correlations with outcomes such as mortality and assess their contributions to the study results.

The COVID-19 pandemic has had far-reaching effects on health-care systems and research globally. However, in our study, we found that the COVID-19 pandemic did not have a substantial impact on the results and conclusions. This is mainly attributed to the fact that the study was conducted in a city that was not severely affected by the pandemic during the study period. The prompt and effective measures implemented by the government helped contain the spread of the virus, allowing the study to proceed without significant interruptions. During the study period, the city where our research took place, Kunshan City, experienced very few infections. Therefore, we can confidently state that the COVID-19 pandemic did not influence our study.

A recent study has shown that administering oral CHO before surgery can significantly reduce the LOS by 1.2 days in patients who underwent intramedullary nailing surgery for proximal femur fractures [18]. Patients undergoing primary total knee arthroplasty did not experience this effect [48]. Our research also revealed that the administration of oral CHO before surgery significantly improved both the LOS and hospitalization costs. Consistent with the previous research, the results indicate that preoperative oral CHO can reduce postoperative complications and the need for additional medical interventions [49–51], resulting in cost reductions for both patients and healthcare systems.

It is important to note that the outcomes may differ based on the surgical technique and patient type, and additional study is required to establish the ideal time and dosage of oral CHO to maximize its efficacy and safety. Nonetheless, our study highlights the potential benefits of administering preoperative oral CHO to improve outcomes of elderly patients undergoing orthopedic surgery for OPFs and reduce health-care costs. This approach should be considered a promising strategy for enhancing recovery after surgery and improving patient satisfaction.

We decided to exclude patients with a high CCI (> 2) from our analysis to reduce the potential confounding influence of severe comorbidities on the interpretation of our findings. The CCI is a verified scoring system that considers a variety of medical disorders as well as their impact on mortality. We were interested in concentrating on the specific intervention or factor being evaluated by eliminating patients with a high CCI and limiting the influence of comorbidities that could independently affect the outcomes of interest by excluding patients with a high CCI. Research studies that target particular outcomes, including mortality, usually involve the exclusion of patients with a high CCI. This procedure lessens the possibility of bias from severe comorbidities, improving the study's internal validity. Nevertheless, it is crucial to understand that utilizing this exclusion criterion may impose restrictions on the applicability of our results to patient cohorts characterized by higher levels of comorbidity. Potential directions for future research could involve investigating the possible effects of incorporating patients with a high CCI and examining the potential interplay between comorbidities and the carbohydrate intake intervention currently under consideration.

Our choice to exclude patients with diabetes from our study holds significant significance for interpreting our findings and understanding the underlying mechanisms of preoperative oral CHO consumption. Diabetes is recognized for its impact on glucose and insulin metabolism, and it is commonly linked to the presence of chronic inflammation [52, 53]. Our study specifically targeted a cohort that did not have pre-existing glucose dysregulation or systemic inflammation by omitting diabetes patients. The decision to exclude persons with diabetes from our research enabled us to focus specifically on the immediate impacts of preoperative CHO intake within a more uniform group. However, this approach restricts our results' applicability to the broader elderly population undergoing orthopedic surgery for OPFs, encompassing individuals with diabetes. In order to further our understanding of the potential relationships between carbohydrate intake, glucose/insulin metabolism, and inflammatory status in diabetic patients, it is recommended that future studies incorporate individuals with diabetes into their research.

The current study has various advantages, including PS matching to limit the impact of confounding factors. However, there are numerous limitations to consider. Firstly, it is crucial to acknowledge that our study was conducted retrospectively, potentially introducing unmeasured confounding variables that may have impacted our results. Our analysis did not consider potential confounders, such as the duration of surgery and inflammatory markers, due to incomplete data. These variables have the potential to be crucial variables in the study. Secondly, since only one institution participated in the study, the results might not apply to other contexts. Furthermore, it is essential to understand the substantial limitations of a small sample size of deaths within 60 days, which amounted to 26 cases (*N* = 26). The small number of fatalities could influence our research outcomes' statistical power and generalizability. Given the restricted number of occurrences, it is necessary to exercise caution when interpreting the findings. Additional research is required to confirm and enhance our findings' validity, particularly by including a bigger sample size and a broader range of events. Finally, given the likelihood for CHO intake to influence variables such as functional recovery and quality of life, the study did not evaluate the long-term results of the patients, which may be significant.

## Conclusions

The outcomes of this study indicate that older individuals undergoing surgical therapy for OPFs may benefit from consuming oral CHO before their orthopedic surgeries, resulting in better outcomes and lower mortality risk. These findings have the potential to inspire the development of therapies aimed at improving outcomes in this patient population and could have significant implications for clinical practices. To validate these results and better understand the underlying mechanisms, further extensive research is required.

## Data Availability

The datasets used and/or analyzed during the current study are available from the corresponding author on reasonable request.

## Abbreviations

CHO: Carbohydrate
OPF: Osteoporotic fracture
PS: Propensity score
LOS: Length of hospital stay
PONV: Postoperative nausea and vomiting
RCT: Randomized controlled trial
OPFRS: Osteoporotic fracture registry system
AKHJU: Affiliated Kunshan Hospital of Jiangsu University
CCI: Charlson comorbidity index
BMI: Body mass index
SD: Standard deviation
CI: Confidence interval
ASA: American Society of Anesthesiologists
AST: Aspartate aminotransferase
ALT: Alanine transaminase
OR: Odds ratio
VIF: Variance inflation factor
LRT: Likelihood ratio test
HLA: Human leukocyte antigen
